# Social prescription in the US: A pilot evaluation of Mass Cultural Council's “CultureRx”

**DOI:** 10.3389/fpubh.2022.1016136

**Published:** 2023-01-19

**Authors:** Tasha L. Golden, Alyson Maier Lokuta, Aanchal Mohanty, Alyssa Tiedemann, T. W. Cherry Ng, Maanasa Mendu, Nicole Morgan, Maria Nagae Kuge, Tessa Brinza

**Affiliations:** ^1^International Arts and Mind Lab, Johns Hopkins University School of Medicine, Baltimore, MD, United States; ^2^Center for Arts in Medicine, University of Florida, Gainesville, FL, United States; ^3^New Jersey Performing Arts Center, Newark, NJ, United States; ^4^Harvard College, Cambridge, MA, United States

**Keywords:** social prescription, arts and health, community referrals, wellbeing, social determinants, social care, health care, public health

## Abstract

**Introduction:**

As the field of public health strives to address the impacts of social determinants of health, it has seen increasing interest in community-referral practices that expand health care beyond clinical spaces. However, community arts and culture organizations are rarely included in these practices, despite accumulating evidence of associated health benefits. In addition, such inclusion has not been formally studied. In response, this article offers an evaluation of “CultureRx” in Massachusetts (MA): the first US model of arts on prescription. The program is a partnership between 20 healthcare providers and 12 cultural organizations, in which providers can offer “prescriptions” to cultural experiences to support patients' health.

**Methods:**

Evaluation was undertaken to illuminate participant experiences, program successes and barriers, and recommendations for further development. The cultural organizations collected participant data (*n* = 84) and completed surveys about their own experiences (*n* = 12). Authors conducted semi-structured focus groups and interviews with healthcare providers (*n* = 33). Data analysis was customized for each dataset.

**Results:**

Findings indicate that participants enjoyed and hoped to repeat their prescribed experiences, which they saw as beneficial to wellbeing. Providers identified the program as a new and critical addition to their toolkits; they also indicated it had a positive effect on their own wellbeing. Cultural organizations reported varied challenges, learnings, and recommendations.

**Conclusion:**

The CultureRx pilot suggests that integrating arts/culture assets into health and social care approaches can enrich and improve traditional US models of community referral. By including arts/culture resources when addressing social determinants of health, communities will be better positioned to equitably and holistically advance health.

## 1. Introduction

### 1.1. Background

The field of public health recognizes that health is not determined strictly or even primarily by medical care, nor is it mainly driven by individual behaviors and biologies. In fact, “[m]edical care is estimated to account for only 10–20 %” of contributors to health (1)–with up to 50% determined by environments and socioeconomic factors (2). These contextual influences are referred to as “social determinants of health” and traditionally include affordable and stable housing, healthy food, employment, quality education, transportation, safety, and clean air and water, sociocultural norms, and political, social, and financial capital (3, 4). Increasingly, social determinants are also understood to include arts, culture, and nature (5–7). Differential experiences with social determinants result in health disparities and inequities, to the extent that one's zip code can determine one's life expectancy (8, 9). The more we understand about the effects of social determinants of health, the clearer it becomes that traditional healthcare practices cannot themselves protect or improve human health. A more holistic approach is urgently needed.

In response, current efforts to improve social determinants include advocating for health impacts to be considered in *all* policies (3, 10, 11), developing more community-based strategies that address health, and honoring how communities themselves cultivate health–often drawing upon cultural identity and grassroots organizing (12–14). In addition, calls have been made to link healthcare with community practices, including arts/culture, to collaboratively promote health (15–17). While such efforts show promise, further innovation and collaborative approaches are needed to move the needle on health and health equity.

### 1.2. Social prescribing

Related to efforts to address social determinants of health, the United States (US) is witnessing growing interest in a model of care called “social prescribing,” first practiced in the United Kingdom (UK) in the 1980s. Though lacking an official definition (18), social prescribing can be broadly understood as using community-based services to address non-clinical and subclinical needs. In this model, patients can be referred by providers to resources ranging from housing and food assistance to job and skills training, volunteering, human-animal interaction, time in nature, and arts/culture activities such as dance classes, museum visits, musical performances, etc. (19–22). Use of services are free to those with a “prescription.”

### 1.3. Community care in the US

Referral practices such as social prescribing are part of core healthcare processes in many countries (23) and related programs and practices are under study in additional regions (24–26). In the U.S. social prescription is only now being piloted (see “Section 1.4”) though similar approaches have long been practiced. For example, many US providers refer patients to local organizations for assistance with housing, education and job training, transportation and food needs, rehabilitation services, support groups, and more (27–32). Like social prescription, typical community referral networks address social determinants of health, are necessarily local/regional, and typically focus on populations facing financial need, mental health concerns, homelessness, or substance use.

As a departure from social prescription's focus on prescribers as a hub of care, many US referral networks function multi-directionally: with various entities in the network referring to one another (33, 34). US networks also differ from social prescribing in that they rarely formally include resources related to arts/culture activities, volunteering, or time in nature, despite growing evidence of these experiences' impacts on health, recovery, and quality of life. The addition of such resources to existing networks remains a largely unexplored–and highly promising–path for improving community health.

### 1.4. “CultureRx” in Massachusetts

CultureRx is the first formal social prescription program in the US to incorporate arts and culture prescriptions. It was launched in 2020 by Mass Cultural Council (MCC), a Massachusetts (MA) state agency that promotes the arts, humanities, and sciences to foster the cultural life of residents *via* grants, initiatives, and advocacy efforts. MCC's CultureRx initiative allows healthcare providers to “prescribe” community-based arts/culture experiences that support patients' or clients' health. It was implemented to advance the wellbeing of MA residents and to provide a US model for improving community health *via* increased access to arts/culture. A timeline for CultureRx implementation and evaluation is offered in Table 1. The program provides cultural organizations with funding to 1) develop/sustain partnerships with healthcare providers, and 2) cover costs of prescribed services. Its first two phases were limited due to the COVID-19 pandemic; however, in-person gatherings became feasible as Phase III began. Thus Phase III provided the first full pilot of the program, and MCC engaged a lead consultant and four-person advisory task force (ATF) to conduct an evaluation. The resulting study, documented below, identifies barriers and opportunities while illuminating participant outcomes and promising practices. It is intended to inform additional programs and arts-health integration practices across the US.

**Table 1:** “CultureRx: social prescription” program timeline.

**Phase I Throughout: advisory meetings and trainings for grantees**	**October 2019**	**Limited application opened for cultural organizations to participate in the CultureRx pilot**
	December 2019	Eight cultural organizations selected, which are partnered with 2 healthcare facilities Each grantee receives $2000 to support their CultureRx program, with additional funds available to reimburse filled prescriptions
	January 2020	Phase I implementation begins
	June 2020	Phase I ends; final grantee reports submitted
**Phase II** Throughout: regular advisory meetings and trainings for grantees	September 2020	To expand project statewide, application is opened for cultural organizations across MA to participate in CultureRx
	October 2020	12 grantees selected, which are partnered with 20 healthcare providers/facilities Each grantee receives $5000 to support their CultureRx program, with additional funds available to reimburse filled prescriptions Phase II implementation begins
	June 2021	Phase II ends; final grantee reports submitted
**Phase III** Throughout: regular advisory meetings and trainings for grantees	September 2021	Phase II cohort invited to reapply for participation, this time with $10,000 provided upfront (no reimbursement) Evaluation Consultant and Advisory Task Force (ATF) brought in to develop pilot evaluation
	October 2021	Organizations notified of award
	November 2021	Evaluation Consultant and Advisory Task Force members interview cultural organizations to determine appropriate outcomes and data collection processes
	December 2021	Customized evaluation plans distributed to each cultural organization
	January 2022	Phase III implementation begins Data collection begins
	April 2022	Focus groups held with healthcare provider partners Evaluation survey administered to cultural organizations
	May 2022	Participant data collection ends; data submitted to evaluation team
	June 2022	Phase III ends; final grantee reports submitted

## 2. Methods

### 2.1. Population

Data were collected from participants across 12 cultural organizations (*n* = 84) ranging in age from 4 to 90. Data were also collected from staff representing the 12 cultural organizations (*n* = 21), as well as healthcare providers with whom those organizations partnered (*n* = 33). For the latter two groups, demographic data were not included.

### 2.2. Participant data collection

The 12 cultural organizations offering services through CultureRx served varied populations and geographic communities, with varied goals related to the health and wellbeing of participants. Similarly, their healthcare-provider partners ranged from community-based healthcare practices to counselors and social workers to a children's hospital, resulting in varied health objectives, referral needs, and tracking processes (see Table 2). In short, though all partnerships were part of a single initiative, they required distinct considerations regarding evaluation. In addition, given that many arts experiences are intended to be immersive or intimate, data collection processes risk affecting the experiences they seek to assess. Considering these factors, evaluators created customized evaluation plans that were responsive to each organization.

**Table 2:** Cultural organizations and their healthcare partners.

**Cultural organization**	**City**	**Healthcare provider partner(s)**
Berkshire Theater Group, Inc.	Pittsfield	MACONY Pediatrics and Community Health Programs
Community Access to the Arts, Inc.	Great Barrington	MACONY Pediatrics and Community Health Programs; State Department of Developmental Services (DDS); UCP of Western MA; Berkshire County Arc (BCArc); Volunteers in Medicine; Family Support Center
Community Music Center of Boston, Inc.	Boston	Local community health center; individual practitioners
Community Music School of Springfield, Inc.	Springfield	Caring Health Center (CHC); Latino Counseling Center
Commonwealth Zoological Corporation	Boston	Harvard Street Neighborhood Health Center
Enchanted Circle Theater, Inc.	Holyoke	Caring Health Center (CHC)
Massachusetts Audubon Society, Inc. (Mass Audubon)	Lincoln	MACONY Pediatrics and Community Health Programs; Volunteers in Medicine
Massachusetts Museum of Contemporary Art Foundation, Inc. (Mass MoCA)	North Adams	MACONY Pediatrics and Community Health Programs; Department of Children and Families (DCF)
Museum of Fine Arts	Boston	Child Life Services at Boston Children's Hospital
The Norman Rockwell Museum	Stockbridge	MACONY Pediatrics and Community Health Programs
Sterling and Francine Clark Art Institute (The Clark)	Williamstown	11 individual and group mental health practitioners
Urbanity Dance, Inc.	Boston	Sargent College of Health and Rehabilitation Sciences

Plan customization began with Zoom interviews with lead staff from each cultural organization that was involved with CultureRx programming. Interviews were attended by an Advisory Task Force (ATF) member and led by the evaluation consultant using a semi-structured facilitation document. Organizations were asked about their program and its goals, the health outcomes they saw their program affecting, barriers to participation, how they had previously assessed their work (and how it went), concerns about evaluation, and what they would like to do or change in the coming year. Evaluators combined interview data with Phase II final reports, and developed 12 custom plans to assess participant experiences. Organizations used these to collect data from their participants.

### 2.3. Healthcare provider and cultural organization data collection

To gather data from participating healthcare providers, six focus groups and three one-on-one interviews were conducted by the evaluation consultant and attended by an ATF member who is a healthcare provider. Focus groups were 40–60 min in length, with 3–7 healthcare providers in attendance; interviews were 20–35 min. A semi-structured facilitation guide was used with questions regarding overall impressions, program usage, anticipated participant outcomes, logistics, and recommendations. Meetings were recorded with permission, then transcribed and de-identified for analysis.

Data from cultural organizations were obtained *via* a Google Forms survey that inquired about participation and data collection during Phase III. It also included open-ended prompts regarding program successes, challenges, and recommendations. Qualitative data were transferred to a word processing application for analysis.

## 3. Analysis

Data analysis was conducted by a team of eight researchers representing multiple institutions and disciplines including public health, psychology, arts, and neuroscience, among others.

### 3.1. Participant data

Basic descriptive statistics were derived from all quantitative data. For qualitative data (open-ended responses), an evaluation researcher reviewed each dataset, selected customized methods of analysis (e.g., narrative summaries, word counts, thematic analysis), and confirmed these with fellow researchers.

### 3.2. Cultural organization and healthcare provider data

A thematic analysis was conducted of qualitative data from both cultural organizations and healthcare providers. To begin, a research team member developed preliminary codebooks for the healthcare provider focus groups and the cultural organization surveys. The full research team was then divided into pairs, which were assigned 2–3 focus group transcripts and two documents from the surveys. Each pair conducted an initial round of coding, identifying missing or overlapping codes. The team then convened to discuss needed codebook edits; this resulted in a focus group codebook with 13 codes, and a survey codebook with 12 codes. The pairs used the updated codebooks to conduct additional rounds of coding. For each code, exemplar quotes were compiled, and frequency counts were generated to help identify additional patterns (35). Finally, the team convened to identify themes.

This study was reviewed and approved as exempt research by Johns Hopkins Medicine's Internal Review Board. All focus groups were recorded with permission. In addition, information about the cultural organizations and healthcare providers engaged in CultureRx is publically available in a published report and on MCC's website.

## 4. Results

### 4.1. Initial cultural-organization interviews

Table 3 provides an overview of the health outcomes that cultural organizations expected or designed their programs to address, reported during initial interviews and optionally modified.

**Table 3:** Expected health outcomes.

**A. Organization**	**B. Anticipated outcomes, November 2021**	**C. Changes/Additions, April 2022**
Berkshire Theater Group	Mindfulness, trust/comfort, sense of connection or belonging with community, family memories, joy, break from everyday stress	
Community Access to the Arts, Inc. (CATA)	Social connection, occupational health, safety and inclusion, peer support, creative expression	Access to additional opportunities for day programs and other scheduled activities
Community Music Center of Boston, Inc. (CMCB)	Ability to pursue interests, self-efficacy, self-esteem, sense of connection/belonging, safety	Sustained connection with CMCB over time
Community Music School of Springfield, Inc. (CMSS)	Support for mental and physical health, connection with one's culture/cultural identity, social connection, calm/mindfulness, pause in busy life	Vaccine confidence and uptake of other health information that is provided at program events or via program communications
Commonwealth Zoological Corporation	Family togetherness, positive memories, keep up with health screenings, mental health of new mothers	Mental health for all patients, time in nature, physical movement
Enchanted Circle Theater, Inc.	Sense of community/connection, creativity, self-expression, mental health	
Massachusetts Audubon Society, Inc. (Mass Audubon)	Mental health and stress levels, physical health, sense of inclusion/welcome, use of additional services/experiences	
Massachusetts Museum of Contemporary Art Foundation, Inc. (Mass MoCA)	Quality of life, safe place for families to interact, trusted connections between families and social worker(s), mental wellbeing	
Museum of Fine Arts (MFA)	Social engagement/connection, mood/mindset, decreased pain, positive staff impacts, connection with community	
The Norman Rockwell Museum	Social connection, enjoyment/relaxation, dialogue, sense of connection with community	Structured opportunities for family interaction
Sterling & Francine Clark Art Institute (The Clark)	Support for mental health therapy, increased access by broader community	Expanded sense of the types of places one can engage with and belong
Urbanity Dance, Inc.	Mobility, balance, strength, daily physical functioning, peer support, mental health, quality of life, meaningful time with others	Decreased stigma regarding varied bodies and abilities

### 4.2. Evaluation implementation

The January 2022 pandemic surge caused implementation delays for most organizations. For some, programming and data collection remained out of reach throughout Phase III. Of the 12 cultural organizations, eight provided data for evaluation. An overview of participation is offered in Table 4.

**Table 4:** Culture Rx participation overview.

**CultureRx participation**	
Total number of referrals	414
Total number of participants	363
Total number of participants from whom data were collected	84
Number of additional referrals anticipated before June 30, 2022	381

Data collection periods varied by organization, with four providing 12+ weeks of data; one providing 4–7 weeks of data, and three providing 1–3 weeks of data. Remaining organizations did not collect data.

Most cultural organizations (58.3%) reported having applied their evaluation plan either exactly as intended or with modifications. The remainder was unable to collect data. Given the evaluation's emphasis on aligning with organizations' goals and processes, the survey inquired about this alignment. 83.3 percent reported that their plan aligned with their process as an arts/culture organization. The remainder faced implementation challenges due to program timing or a lack of participants.

### 4.3. Participant data

For organizations that collected participant data, results are provided in Table 5.

**Table 5:** Participant data.

**Berkshire Theater Group**	Berkshire Theater collected three responses from children and two from parents, ranging in age from 10-40. Using a 10-point Likert scale, participants were asked to respond to various statements about their experience. Responses were provided to the following: “I felt safe and welcome here” (***n =*** 5; mean rating 9.6); “I want to come here again” (***n =*** 4; mean rating 10); “I made a positive memory with the people I came with” (***n =*** 1; rating 10).	
**The Clark**	The Clark's participant surveys were developed by the organization itself, as a continuation of multiple years of assessment. The Clark received 11 unique responses; visitors were asked to respond to two questions using a five-point Likert scale: “How was your experience at the Clark today?” (*n =* 11; mean rating 4.55), and “How much did your visit enhance your well-being?” (*n =* 11; mean rating 4.32). The Clark's surveys provide an option to offer additional feedback; multiple participants reported having “fun” at the museum; other descriptors included “inspiring,” “calming,” and “just what I needed.” One participant indicated that the experience had helped them learn that they can “overcome a lot of stuff.” They reported that seeing “the wind…blow a lot of stuff away…gave me confidence.” Another stated that “the fresh air, the sunshine, the views, a safe space did [their] soul good” and they “…cannot wait to come back.” Though this individual had grown up in the Berkshires, this was only their second visit to the Clark and they reported being glad that CultureRx “put it back on [their] radar.”	
**Community Music Center of Boston (CMCB)**	CMBC had two CultureRx participants, aged 9; each took eight music lessons. They were asked at the end of each session how they were feeling about the lesson and their progress. Participants tended to respond positively, even if they also reported being tired or confused. Both mentioned having fun and liking to play piano.	
**Community Music School of Springfield (CMSS)**	CMSS had three participants; surveys asked them to rate statement responses using a 10-point scale ranging from “Not at all” to “Very Much.” Responses were provided to the following: “I feel welcome and included here” (*n =* 3; mean rating 9); “This program has improved my mental health” (*n =* 3; mean rating 5.7); and “I want to come back and keep participating” (*n =* 3; mean rating 9). Participants were also asked if they made new friends in the program; one responded “Yes.”	
**Mass Audubon**	Mass Audubon had two participants; both “strongly agreed” they felt safe and welcome as a visitor to a Mass Audubon Wildlife Sanctuary. Both responded “Yes” when asked if they plan to visit again, and both reported that it was not challenging to access the sanctuary.	
**Museum of Fine Arts (MFA)**	The MFA had 48 participants in their program at Boston Children's Hospital, ranging in age from 4-17. Most art sessions in the hospital are one-on-one, and participants are asked questions at the end of the visit. When asked “Would you like to do this again?” 92% participants responded yes and 2% replied maybe; data were missing for 6%. Facilitators also recorded notes about interactions with participants, generating qualitative data on which a thematic analysis was conducted. Four themes emerged, documented below:	
	**Theme**	**Description**
	**Patient experiences**	A range of experiences documented using codes “pre-arts engagement” (what patients were doing before the session began), “change in affect,” “change in reaction (to the art session),” and “patient wants to engage.” Even when patients were not feeling well, they still often said they wanted to make art, or asked the facilitator to leave art kits so that they could make art later. This indicates the value of tangible materials that can be left with participants.
	**Positive experiences**	Indications of interest, such as when patients created multiple projects or asked to do more. Actively making art (painting, coloring, sculpting, using Model Magic, drawing, tie dye, and collage-making) was the most appreciated aspect of participants' experience; a combination of “painting and talking” emerged as most popular.
	**Family involvement**	Reports of patients' family members (typically parents) engaging in the arts session with the patient, and/or assisting with communication between facilitator and patient. Some facilitators reported benefits of the session for family members
	**Factors affecting the art session**	Immediate environment (noise levels; people going in and out), pre-existing interest in art, recent receipt of difficult news, patient limitations. Personal connections also affected sessions, appearing as important as activities themselves (many patients were “chatty;” facilitators often painted alongside the patient or helped them incorporate art into various forms of play). Findings suggest familiarity and connection were helpful to patients and played a role in their enjoyment.
**Norman Rockwell Museum**	Norman Rockwell had four participants, including children and caregivers. Three responded to questions using a 10-point Likert scale, while one child answered with Yes or No. Questions included, “I felt safe and welcome here” (mean rating 10); “I'd like to come again” (mean rating 10); and “Being here made me feel better” (mean rating 9.7). The child responded “Yes” to all questions. Participants were also asked what their favorite part of the visit was; responses included “Seeing the art” and “Learning new things.”	
**Urbanity Dance**	Urbanity held 16 classes designed for individuals with Parkinson's Disease; attendance ranged from 1 to 7 participants, with ages ranging from 60-90 years. Attendees were asked at the end of each class to respond orally to the question, “How do you feel now compared to when you came in?” This approach accommodated those for whom writing and screens posed challenges, and provided a way to nurture social connections among participants. In all, Urbanity Dance participants reportedly find the class helpful both physically and mentally. They also appear to feel comfortable sharing a range of experiences, indicating group trust and connection. Responses fell into four categories:	
	**Physical descriptors**	Respondents frequently reported feeling “looser,” “more limber,” and “less stiff;” they also reported feeling achy “but in a good way.” Others reported some pain (e.g., an aching knee), or feeling warm or warmed up.
	**Energy levels**	Reported changes in energy levels included being “way more energized,” “more lively,” “ready to get up and go,” relaxed, “exhausted” from the physical activity, or ready to “handle anything.”
	**Mental and emotional changes**	Gratitude was a common response; descriptors also included calm, centered, stimulated, accomplished, and “less scattered.”
	**Other**	Some responses potentially fit multiple categories; e.g., feeling “hipper, relaxed and swinging,” “free-floating,” “stylin,” or “free.”

### 4.4. Cultural organization surveys and provider focus groups

During thematic analysis, 19 codes were established. Eight themes emerged: Participant Experience, Provider Experience, Cultural Organization Experience, What Went Well, Barriers, Evaluation, Short-Term Recommendations, and Long-Term Recommendations. Themes are detailed below.

#### 4.4.1. Participant experience

This theme encompassed feedback regarding participant experiences and outcomes; findings have been divided into anticipated and reported benefits and experiences.

##### 4.4.1.1. Anticipated benefits/experiences

This section describes experiences that a given arts program was expected or designed to address. Multiple providers viewed arts/culture-based activities as positive motivating forces given the potential for enjoyment, social or community connection, or novel experiences. For example, a physical therapist working with individuals with Parkinson's disease perceived classes at Urbanity Dance as helping patients remain engaged by physical activity, which is essential for their health. Mental health counselors and pediatricians reported making CultureRx referrals to clients or patients who don't “have a purpose for their day” or “just don't have motivation” to leave their home.

Providers also viewed arts/culture programs as tools to help clients/patients improve their sense of connection with themselves, family members, or their community. A physician said that CultureRx gives families an “opportunity to do something that they normally wouldn't do, and have them be able to share that and learn from it.” Another mentioned refugee families' need to feel connected with their new communities, and argued that free, welcoming experiences at arts/culture institutions could provide such connection. Similarly, a therapist noted that some clients “are struggling and having a hard time connecting with who” they are or want to be, and prescribed a day at The Clark to help reset and reconnect.

Several providers saw arts/culture activities as helping address trauma, self esteem, and other challenging life experiences. At Boston Children's Hospital, healthcare providers described visits from MFA artists as positively altering children's experience of being in the hospital: “[T]hey can know that every time their door opens, it's not [always] someone coming in to do something medical…they're just here to do something fun, which is…kind of unique.” In addition, when facilitators offer MFA tickets to children and their parents, it “gives [parents] a little bit of hope of like…'We will get out of here, one day, and we can go to the MFA and…still have this great experience.” Similarly, pediatricians and mental health clinicians reported referring “people who are sort of depressed or anxious,” “can't get out of their own way or their own head,” or who had “difficulties with self-esteem and with expressing their emotions verbally.” Mental health providers added that the program is helpful as an alternative to traditional therapy approaches: “[P]eople don't necessarily want to go meet a new person and have to tell their story all over again and that...kind of limits some people from wanting to engage in therapy. So this is an alternat[ive].”

Finally, providers mentioned the value of offering an experience that was simply about enjoyment, “beauty,” or responding to their interests. A physician noted that the CultureRx program helps “tap into [patient] strengths…so that they don't go to crisis and they aren't actually getting to the place of needing the other supports…”

##### 4.4.1.2. Reported benefits/experiences

Some cultural organizations described seeing participants connect with others (e.g., parents connecting with children *via* a museum exhibit), or witnessing improvements in mood. Others heard participant feedback about physical and mental changes, with Urbanity Dance sharing, “It is incredibly affirming to hear that the students' bodies are overall feeling more mobile, flexible, and ‘better' [and] that there is an expressed emotional and community uplift.”

Several mental health practitioners reported observations regarding their weekly clients, with one noting that a day alone at a museum had helped a client reconnect with an important life interest. Another said that a client's interaction with a particular museum piece had supported discussions in therapy; a third said clients had been sharing “where they felt like they saw themselves in the [art] pieces, which seems like it's felt very empowering.” Similarly, a provider mentioned that discussing art had offered an opportunity to “affirm that what [their client] was drawn to was good and right and fine.”

Providers at Boston Children's Hospital also reported patient experiences with MFA's art sessions. One described a patient who “really looked forward to” the arts-activity visits: “It was an exciting part of her week…It just changed her mood.” Another mentioned how thankful patients were to log onto virtual sessions during the pandemic, particularly since many other activities were put on hold. Similarly, a provider described a “little girl who's been here for months” ‘who “was just really sad” when she was unable to see the MFA facilitator–indicating the significance of that patient's connection with the artist. More generally, providers mentioned that long-term patients “look forward to [MFA] activities.”

Healthcare providers also shared that arts experiences positively affected self-esteem and self-efficacy, such as by allowing people to explore new interests in a safe way. Providers mentioned patients building a “sense of mastery and success” at Urbanity Dance classes, having a sense of pride in the art they produced at CATA programming, and “finding new sources of confidence” through CMBC music classes. These changes often encouraged participants to continue engagement.

Finally, several providers reported positive patient responses upon receiving a CultureRx prescription, such as “pleasure and the delight on the faces of moms and their children.” A physician described a patient who exclaimed, “That was like the best doctor visit I've ever had in 72 years. Like it was so fun and I get theater tickets!”

#### 4.4.2. Provider experience

Providers described referring clients or patients to CultureRx opportunities as very different from their typical referrals. A physician said their recommendations are usually about taking things away (e.g., reducing caffeine) or adding things that demand time and effort (e.g., organizing a family outing). By contrast, CultureRx allowed them to prescribe something “enjoyable” or “just like, *fun*” that patients could readily participate in. “It feels like you can give something to people and it's just nice and it makes people happy,” a physician shared. “I feel like we don't do a lot of making people happy in medicine.” Another provider said they wanted to make these opportunities “more accessible to all community members.” Contrasts with typical practices was a common theme, with CultureRx offering “such a value-added experience” for providers and patients alike.

Providers also mentioned the value of adding non-clinical experiences to their work. A mental health provider noted that the arts-based activities provided “a great link [or] jumping off point of creating conversation where you can take [our work] a little bit deeper.” Notably, some providers indicated that their own experience with a CultureRx activity influenced their referrals: “Once I [had personally attended] a class, then it was 5 million times easier to recommend it to my patients.”

In addition to benefiting their clients/patients, several providers felt the CultureRx program supported their own wellbeing and work experience. “[I]t's really fun to give out these prescriptions,” a physician shared, linking their own enjoyment to “the family's reaction–I mean…they're very appreciative...they're excited.” Another stated that providing CultureRx referrals gave them “a lot of joy.” Most providers had never before been able to refer their patients/clients to arts-based experiences, and doing so was impactful: “[I]t feels like prescribing beauty in your life,” a physician stated. “I've never had a chance to do that, but I feel like that's kind of what this is. And of all of Maslow's hierarchy of needs, beauty in your life seems like it should be on every level, wherever people are… [CultureRx] allows for that to manifest.”

A few providers noted that before CultureRx, they could *suggest* arts/culture-based activities to clients/patients, but follow-through was minimal. In their view, there is a significant difference between *suggesting* that patients engage with activities or interests and “providing a means in which they can actually” *do it*.

#### 4.4.3. Cultural organization experience

Emerging parallel to “Provider Experience,” this theme encompassed organizations' experience with CultureRx, including impacts on their program. It overlaps significantly with *Barriers* and *What Went Well* (see below), with experiences generally reported as positive and a source of growth or learning: “CultureRx is an incredible program, and every edit, tweak, adaptation brings us closer to creating an efficient and successful program.” Organizations reported that their involvement with CultureRx had led to more engagement with new populations, and with their communities in general. For example, The Clark reported an increased sense of being a “caring community player.”

#### 4.4.4. What went well

##### 4.4.4.1. Increasing awareness and expanding care options

Most generally, healthcare providers reported that they were glad to be able to offer patients/clients something that went beyond traditional models of care. Another successful aspect of CultureRx was its ability to increase awareness of and access to community arts. One provider stated that before receiving a referral, a “lot of people don't even realize that they can go to these places.” Increasing patients' awareness and access was a motivating factor for prescribers, with some acknowledging the power of a doctor's recommendation to drive engagement and the importance of providing access to experiences that are otherwise out of reach.

##### 4.4.4.2. Partnerships between healthcare providers and cultural organizations

Communication went well for many CultureRx partnerships. Some providers praised their cultural-organization partners for communicating consistently, and several complimented their partner's responsiveness and adaptability. As one example, Boston Children's Hospital appreciated that the MFA's Artful Healing personnel were “so receptive” and that “they'll listen to us and take our lead…because we can't get into too much detail about what's happening.” It was also helpful when providers had multiple options for patient/client engagement. For example, MACONY is connected to five different cultural organizations; providers found this helpful because it “offers options, so there's a participatory nature in that the person has a choice [and] not just the one thing that they're given.”

##### 4.4.4.3. Flexible scheduling

Different groups saw success with different scheduling formats; for example, Sargent noticed that with Parkinson's patients, having a set time and place for the engagement ensured a higher likelihood of participation compared to an activity that can be done at any time. Other providers noticed the opposite among different populations, noting that flexible scheduling was helpful.

##### 4.4.4.4. Tangible materials and incentives

Tangible, take-home elements also proved to be important. Healthcare providers associated with The Clark and MFA were enthusiastic about the fact that these two organizations provided packets that include art, activities, tickets and, in The Clark's case, a coupon for their cafe. Several mental health providers said that The Clark's packets provide “something to talk about” or use immediately, even if clients do not wind up visiting the museum. More generally, providers emphasized that packets were meaningful in themselves, as they provided a tangible gift that did not rely on in-person engagement.

As a specific tangible component, mental health providers emphasized how important it was that The Clark's packets include a cafe coupon alongside museum tickets. They reported this offer as a powerful incentive for attendance and engagement, and shared stories of how it helped clients envision taking an entire day for themselves, or contacting a friend for a coffee. Despite representing a relatively small gift, providers were emphatic about the value the coupon added to the process.

##### 4.4.4.5. Additional helps

Another aspect that worked well included giving clients/patients specific guidance about what to do or look for when visiting the museum: “[T]here's been the follow through when I gave a very specific ‘assignment.”' Funding and support from Mass Cultural Council were also reported as significant, enabling organizations to reach new audiences and welcome populations that may not engage without a referral. Finally, organizations said the evaluation process was helpful; it had enhanced their understanding of their program, and they could use the data to help describe their work in the future.

#### 4.4.5. Barriers

While participant experiences were reported as overwhelmingly positive, providers and cultural organizations illuminated a range of barriers to engagement and benefits. One such barrier, mentioned by almost every provider, was transportation to arts/culture spaces. A western MA provider noted, “We don't have things like Uber, taxis are unreliable, buses stop running at certain times, and like you might have to take four transfers to get from one town to the other.” Transportation barriers were also reported for specific populations; for example, children's access requires making arrangements with their parents/guardians, which can be difficult. Participants with Parkinson's disease were noted as facing the challenge of being unable to drive to dance classes on their own, with those who walk facing difficulties navigating uneven or slippery sidewalks. When transportation was not an issue, finding parking was also cited as a challenge in urban locations.

Notably, as the pandemic drove activities into virtual formats, “the digital option…helped with the transportation barrier by providing an alternative.” However, moving online presented a new barrier for those lacking digital access or facing technological challenges. One organization regretted that they “do not have the IT personnel to properly assist people.”

Language and literacy also emerged as barriers affecting participation. Several organizations noted that English is the primary language they use, which may impede access for the diverse populations they serve. “Though dance itself is universal,” one observed, “we do use English to guide the exercises.” Another organization mentioned that their “printed materials are currently only available in English, which might be a barrier to participants trying to access this information.” Language was also reported as a potential barrier to simply connecting with and scheduling new participants.

Another barrier involved exclusion and intimidation, such as fears about not belonging in a given space. One provider reported that one of their clients “could specifically voice that it was a question of intimidation. Like, ‘there's going to be all those New Yorkers dressed nice in there, and then I'm going to come in.”' The potential for intimidation was also noted by cultural organizations; one said, “Facilitating a first visit when there is a feeling that one might not be well understood seems to be a barrier, especially when frequenting museums is not already a regular activity...” Urbanity Dance referenced the “stigma that you have to be or look a certain way to be a dancer,” noting that “being intentional about representation helps.” Related to a sense of exclusion, relevance and applicability also emerged as barriers, such as teenage patients being uninterested in theater because “they think it's geared toward the younger kids,” or young families not attending because “they don't know…of kid age-appropriate shows.”

Time was a barrier for providers, arts organizations, and participants. Providers noted it could be difficult to find time during appointments to share how a CultureRx experience could benefit their patient. They also mentioned that CultureRx prescription opportunities can be “lost in the shuffle” if providers are not attuned to preventive approaches to health. A care coordinator who assists clinical staff argued that if “providers really understood the benefit of community resources [...], things would connect better.” For patients/clients, respondents noted that making time for a class or museum visit was a challenge amidst busy lives, and cultural organizations observed that “[i]t takes a long time to foster new and trusting relationships.”

Two providers mentioned that a lack of training and representation at cultural organizations were potential barriers to making referrals. One expressed that the art at their partner institution was mostly created by white male artists, which was “really off putting.” The other believed that cultural organizations do not have “the mental health framework where [clients] would be meeting with practitioners trained in trauma,” which made them “a little nervous about what's going to happen when [my clients] get there.”

Finally, as noted, the pandemic and its effects posed barriers for organizations and providers. In addition to canceled or delayed program implementation, data indicate that some participants did not redeem referrals when proof of vaccination or masks were required. This was a particular issue for individuals with disabilities, many of whom providers said are unable to wear masks.

#### 4.4.6. Evaluation

Cultural organizations commonly characterized their experience with the evaluation process as “gratifying.” One reported that it allowed them to verify “[their] observed experiences that participants tend to feel better during/after our visits” and obtain “specific statistics on how many of [their] participants are really interested in visiting (100%!).” Another shared that their evaluation plan was itself beneficial, because its “questions distilled measurables in a way that felt achievable…given the [participants'] capacities.”

Cultural organizations also reported challenges, such as delayed implementation (which reduced available data), lack of referrals/participants, time and staff constraints, and an inability to reach some participants. Two organizations reported that “[their] programming ramps up in the spring” and “late June” respectively, which meant that most of their participant data were unavailable by the evaluation deadline.

Some cultural organizations reported growth in data collection abilities and practices. One found that providing participants with “a coupon to redeem at the concession stand” if they filled out the survey “resulted in a 100% return of participant surveys.” Others reported that developing and sharing clear expectations about evaluation with their staff and practitioners helped ensure that data collection was a “smooth” and “easy process.” Several cited the evaluation process as one of their primary program successes, with one stating, “[I]t's been great to get data-driven confirmation of what I and our educators know in our hearts – that what we do helps people. We see it in action, but it can be hard to explain to people outside of our programs when they don't see the effects in person.”

Healthcare providers offered fewer comments related to evaluation and assessment, but several acknowledged the benefits of collecting data to track changes before and after participation. One specified that even negative participant feedback is useful, in that it can indicate ways to improve experiences, benefits, and training. Providers also noted that data collection could help increase provider buy-in by bridging gaps in knowledge; one mentioned that data could reassure providers of the “safety” of the referred organization(s), because they would better understand participants' experiences.

#### 4.4.7. Short-term recommendations

Healthcare providers and cultural organizations provided recommendations both for their specific partnerships and for the CultureRx initiative as a whole. To increase engagement and accessibility, providers recommended clearer signage at cultural organizations to help referred clients feel welcome. They also recommended offering scheduling coordination *via* text, and greater variety in class/event times to accommodate varied schedules. Several also mentioned improving language access, and another suggested creating data collection tools in multiple languages.

Providers working with individuals with disabilities mentioned that clear information was needed about how the CultureRx program may affect patients' insurance. Others recommended transparency regarding potential future costs of activities, expressing concern that participants could be “let down if they don't realize this isn't a forever program.”

Cultural organizations recommended placing expiration dates on prescriptions to encourage timely participation. A few providers that are partnered with museums recommended that referral opportunities should include not only visits, but scheduled events at the museum, such as art classes. Specific, facilitated opportunities were seen as supporting increased participation while providing opportunities to connect with others.

Some providers recommended expanding referral pathways, because more patients could benefit from the CultureRx experience than they had initially recognized. They appeared to view this as a needed change on the provider end: “[W]e're the ones that probably should be able to step up a little with just recommending it wholeheartedly.”

Related, a cultural organization recommended developing partnerships with schools to increase referrals to arts/culture activities for school-aged children, and providers echoed this. Providers also indicated that school personnel could provide “additional follow-through” on children's CultureRx referrals, even when referrals are initially generated by healthcare providers. Notably, two CultureRx partnerships already include school-based practitioners or coordinators, confirming the potential for these roles.

Finally, several organizations recommended more training opportunities for their staff, and urged the inclusion of additional staff as well as healthcare coordinators. “Right now,” one respondent noted, “there is a small [group] within our organization that is much more deeply involved [with CultureRx] than others.” Organizations and providers also recommended consistent avenues for discussion and collaboration in order to foster trust and co-create practices that are increasingly beneficial to patients/clients.

#### 4.4.8. Long-term recommendations

General and long-term recommendations have been documented below by providers and cultural organizations.

##### 4.4.8.1. Provider recommendations

Many provider recommendations centered on expanding CultureRx. In the program's current iteration, most providers are linked to only one arts/culture organization; if their patients/clients are not interested in that organization or art form, no other opportunities are available. They therefore see additional organizations as supporting their ability to offer more personalized, salient opportunities. Providers also mentioned the value of offering arts-based experiences on location at the healthcare site, rather than strictly referring patients/clients to other locations.

As another form of expansion, providers recommended developing “pipelines” by which participants can become continually involved in arts/culture activities. One provider drew a distinction between giving a “ticket to someone that's interested in theater” and ensuring that such a person learns about opportunities to participate in upcoming plays. Another envisioned CultureRx as a first step in encouraging a child to “find *their* cultural institute, their home.” In short, providers see value in ongoing arts participation, and want patients/clients to have opportunities for long-term involvement.

Providers also recommended expanding awareness of CultureRx in order to increase participation and benefit. One shared that they themselves had not realized that their clinic's partnership with an arts organization was part of a statewide program: “[I]f patients realized–and if the clinicians and physicians *themselves* realized–that this is actually part of a statewide thing, maybe that makes it more interesting” since it would not be perceived as “just a one off…recommendation.” This clinician had personally seen the benefits of referring patients to their cultural-organization partner, but fellow clinicians had not made use of the opportunity. They suspected this lack of interest was linked to a perception of the partnership as a novelty for limited patients, rather than as part of a larger, research-based model. Other providers noted that increased awareness could also lead to more funding.

##### 4.4.8.2. Cultural organizations

Like healthcare providers, many cultural organizations envisioned expanding CultureRx to reach more participants–often echoing providers' recommendations. For example, they advocated for “engaging more healthcare providers so that there are more prescriptions out in the community,” and expressed interest in co-locating arts with health services, so that “cultural engagement [is] embedded in all aspects of healthcare.” One suggested raising awareness by associating CultureRx with “organizations that [currently] provide essential services and support to our community.” Additional funding was often recommended to create larger, lasting programs.

Cultural organizations also imagined concrete ways to better connect healthcare providers, participants, and organizations. One envisioned “a popular local or national list/database/resource that connects individuals and practitioners to Cultural/Arts Partners… [similar to] Psychology.com.” This was described as offering patients and providers more choices regarding the arts/cultural experiences they could engage with to support health. Another organization imagined improving communication and data collection *via* “a portal similar to a healthcare agency's patient portals, to ensure consistent communication between agency staff/admin and participants, as well as [to] document notes, observations, and evaluations.”

In line with providers' recommendation for longer-term engagement, a cultural organization recommended making connections among program participants, such as a “Culture Buddy System” in which CultureRx participants “can opt in to being paired with someone else that has the same culture pass point.” Finally, organizations frequently noted the need to improve health equity and access. They mentioned the value of “ongoing training” in diversity, equity, and inclusion, and one organization envisioned “an advisory board made up of community members, healthcare providers, and cultural representatives [who] would provide oversight on issues of equity, access, and inclusion, and make recommendations.” Both healthcare providers and cultural organizations stated that ongoing communication between entities will enhance this type of program and its benefits as it grows, and both groups noted the potential value of a formal paid position devoted to sustaining and growing the CultureRx initiative.

The above short- and long-term recommendations have been made available in table form at https://tinyurl.com/culturerxstudyrecs.

## 5. Discussion

Findings of this evaluation indicate increasing interest in and support for the integration of arts/culture opportunities into Massachusetts healthcare referral opportunities. This section discusses immediate findings, resulting shifts in healthcare practice, and next steps in building and sustaining similar initiatives in the US.

### 5.1. Immediate findings from CultureRx

Despite differences in data collected across the 12 cultural organizations, a shared finding was that participants enjoyed the experience or activity, felt welcome and safe, and expressed interest in returning or participating again. Some datasets indicated participants' interest and appreciation even when they felt unwell or tired, suggesting that engagement with arts/culture is a positive addition even in challenging moments and contexts. Many activities appeared to stimulate positive physical and emotional changes, such as reduced stress, greater enjoyment or relaxation, pride or self-esteem, and more energy. In addition, no adverse effects were reported. Taken together, this study's findings indicate that arts/culture experiences offer positive outcomes with minimal to no risk, making them an intuitive addition to healthcare referral practices.

This evaluation also revealed challenges associated with piloting a multifaceted process that engages multiple sectors, and these challenges were exacerbated by a global pandemic. In addition, some organizations experienced staff turnovers that delayed implementation, while others underwent this evaluation during their lighter seasons–resulting in fewer referrals/participants. Nevertheless, organizations' response to the evaluation process was positive, suggesting opportunities to generate more robust data over time. In addition, as similar programs are created and studied around the globe, research practices related to measurement, data collection, and reporting should be shared and potentially standardized–allowing for ongoing learning and comparison across regions (36).

Respondents emphasized the importance of equity, access, and inclusion to realize the potential benefits of the current program. Equity and access concerns were relevant not only to arts/culture organizations, but to the healthcare sector–which is not yet designed to provide equitable access to wellbeing, enjoyment, or connection. Respondents also noted difficulties with scheduling, and the need for platforms that could support more efficient referring practices and data sharing. In the future, such platforms could help track resource usage and health outcomes, and offer detailed information to providers (for example, which resources have trauma-informed staff, children's programming, specific accommodations, etc.). Notably, the importance respondents placed on current challenges appeared to derive from a desire to ensure the program reaches more people and that it grows over the long term.

This evaluation also illuminated provider responses to the integration of arts/culture into healthcare. A common assumption related to social prescribing (and to arts integration more generally) is that providers will not refer to arts/culture experiences until more evidence is available regarding the benefits of such experiences (37–39). However, more evidence already exists than many providers realize, indicating a need for awareness and education. As providers' understanding of benefits and opportunities expand, they are likely to write more prescriptions–thus generating more opportunities to evaluate program impact. Nevertheless, this pilot indicates that providers were *not* skeptical regarding the benefits of arts/culture. Instead, they appeared enthusiastic that these resources could provide additional health support for their patients/clients, particularly related to wellbeing and social connection. While the benefits that providers currently associate with arts/culture experiences are limited (see “Next Steps…,” below), they appeared confident that these benefits are available, without risk of adverse effects. Before CultureRx, they had never had a way to make arts/culture-based recommendations that were readily accessible, and thus saw this program as increasing their care capacity.

### 5.2. Promising shifts in healthcare practice

CultureRx appears to improve and expand concepts of what a healthcare visit or provider relationship can be like. While providers in this study had previously referred patients/clients to non-medical and community-based opportunities, most had never made such referrals based on needs for enjoyment, connection, beauty, mindfulness, self-care, pursuit of interests, etc. Providers reported that for their patients/clients, merely being offered these opportunities was meaningful and exciting–sometimes regardless of whether they filled their prescription. Similarly, at Boston Children's Hospital, the addition of MFA's art sessions meant that a child's hospital room did not have to be defined strictly by fear or medical tests; they could also provide fun, exploration, and connection. Such a shift in hospital experiences could have profound effects on mental health and patient relationships with medical teams.

Another distinct aspect of CultureRx is the direct, free access it offers to community resources. One provider mentioned that before CultureRx, they might have recommended experiences like dance classes or a museum visit, since these events are occasionally free. However, this provider noted that a community's free experiences are never listed in one place, and they change frequently. As a result, if a patient wishes to take up a recommendation, they have to do the work of searching for an opportunity, identifying when/where it's offered for free, and finally attending. By contrast, CultureRx referrals are simple: when the provider recommends an experience, access is free.

Finally, findings indicate that the ability to “prescribe” arts/culture experiences can positively impact providers' own health. Providers reported that they often have to emphasize what has gone wrong or what patients/clients must do differently, and that they lack ways to support patients/clients outside clinical settings. The addition of resources *via* CultureRx, and the resources' applications for wellbeing, enjoyment, and connection, appeared to bolster providers' mental wellbeing and work satisfaction. Notably, this benefit may be unique to CultureRx's approach to social prescribing, given that UK models rely upon link workers (not providers) to coordinate prescriptions (40, 41). In a field facing alarming rates of burnout and turnover (42–45), this is a critical finding: being equipped to help patients cultivate enjoyment and connection is good for providers' health.

### 5.3. Implications for community health

This evaluation study suggests next steps in advancing community health *via* programs such as CultureRx. First, providers will benefit from more information about how arts/culture referrals can be supportive to patients/clients. Providers were enthusiastic about CultureRx and associated referrals with several benefits; however, their understanding of the program's utility was relatively limited. For example, a few providers were most likely to prescribe a CultureRx opportunity if the patient/client explicitly indicated an interest in art. While such interest makes a referral intuitive, reliance on interest neglects the wide range of health outcomes for which arts/culture are applicable. Other providers reported making CultureRx referrals for clients/patients in need of self-care, lacking connections, or needing quality time with loved ones. These uses are supported by previous studies and the current evaluation; however, the literature indicates many additional applications, including general stress reduction (46), decreased anxiety and depression symptoms (47–50), increased creativity (51), improved subjective wellbeing (52), and more.

Another step in advancing health *via* programs like CultureRx is to integrate them with existing community-referral networks. As noted, social prescription models are often unidirectional: with healthcare providers making referrals *out to* community organizations. By contrast, some US community-referral models involve multidirectional practices in which various members of a referral network refer clients to one another. In CultureRx, healthcare's engagement was clearly critical; however, community health would likely be bolstered if cultural organizations were also empowered to make referrals on behalf of their participants to needed community resources (e.g., transportation, housing, mental health care, etc). When considering how to apply arts/culture assets toward advancing individual and community health, it may be more valuable to configure them as newer members of existing community referral networks, rather than strictly as new recipients of prescriptions from healthcare providers.

Thirdly, this evaluation indicates that community health programs would benefit from an expansion of available arts/culture opportunities in order to increase personalizability and choice. Such expansion would also make it more likely that patients/clients find options with which they want to engage long-term–thus extending benefits. Expansion should include co-locating arts/culture activities within healthcare settings, and locating healthcare services within community arts/culture spaces. Though study respondents did not mention the latter, this parallel recommendation could help address concerns related to community access and trust. For example, a future iteration of CultureRx may pilot the availability of health checkups or mental health counselors within trusted arts/culture spaces.

Finally, in addressing community health, it is important to note that a model such as CultureRx may be particularly valuable at a time when mental health resources are urgently needed and increasingly lacking–as they are now in Massachusetts and around the US (53–55). Though arts/culture experiences are not a replacement for therapy or other standard treatments, providers' descriptions of this program's benefits indicate that it could function, foster connection(s), improve healthcare encounters, create moments of joy or beauty, and provide material for discussion with friends, family, and eventual therapists or counselors.

## 6. Recommendations

The authors drew upon findings from this evaluation to offer 11 recommendations to support the design and development of both CultureRx and similar programs throughout the US. These are offered in Table 6.

**Table 6:** Recommendations.

**#**	**Recommendation**	**Description**
**1**.	Integrate arts/culture opportunities into existing referral networks.	Referring patients to non-medical resources and experiences is not new in healthcare; however, the presence of arts/culture *among* those referral options is new. Given this evaluation's findings, we recommend adding such opportunities to existing community-referral networks. Doing so is likely to be welcomed by healthcare providers and other community organizations (see Recommendation 7), and it will increase options for health promotion.
**2**.	Put more funds toward creating robust processes for building and promoting the program.	Grant funds were initially perceived as necessary primarily for reimbursing the cost of the free services prescribes receive. However, reimbursement is only necessary if referrals are made and used. Thus while a program builds, it is critical that funds be allocated to program-building processes such as cultivating and stabilizing partnerships with healthcare providers, promoting the program (to providers, other community agencies, the public), developing robust processes for participant connection and follow-up, ensuring data is collected and documented, addressing access concerns, etc.
**3**.	Expand the number of healthcare providers partnered with each organization.	The low number of referrals to some organizations appears related to the limited number of healthcare providers with which they were partnered. Cultural organizations have the capacity to be partnered with multiple providers, as they are unlikely to receive too many referrals. By contrast, too few may significantly limit the program's reach. To address this, cultural organizations could add provider-partners individually, or co-develop healthcare partners with other organizations. As with MACONY/CHC/UCP, being partnered with multiple cultural organizations gives providers and their patients more options; similarly, multiple provider-partners ensures more engagement for each cultural organization.
**4**.	Expand the number of participating cultural organizations.	Many providers requested additional options for patients/clients. A starting point would be to encourage current organizations to work together, since they would then share provider-partners (This is currently modeled by the five cultural organizations partnered with MACONY/CHC/UHP). In addition, given concerns about equity and access–including relevance and cultural responsiveness–we recommend ensuring that smaller, grassroots arts organizations are sought for participation. Such organizations may have significant meaning for specific communities, yet may not have ready access to typical grant opportunities.
**5**.	Design a website and one-pager to help healthcare providers quickly recognize the varied health benefits of each program.	Providers found CultureRx beneficial, but demonstrated a limited understanding of the varied ways in which referred opportunities could support patients/clients. In response, cultural organizations should generate a concise, research-based summary of the ways in which their program(s) may benefit participants (As one example, see Canada's PaRx program website). Funds should be set aside to both research these program-benefit descriptions and make them readily accessible.
**6**.	Consider alternative activities and schedules.	Some participants do better with fixed-schedule events such as classes, while others respond well when they can attend whenever they would like. Whichever scenario is most common for a given organization, we recommend they imagine how they might complement it by occasionally incorporating an alternative option.
**7**.	Pilot the use of digital platforms to link cultural organizations with healthcare providers and other social/community resources.	A growing variety of digital community-referral platforms are supporting community health by providing structures for referrals and community engagement. Many utilize screening processes related to social determinants of health, identify relevant potential supports, and then provide tracked referrals to services (e.g., to transportation, mental health, shelters, etc.). Reports from these platforms indicate parallels with this evaluation's findings, such as (1) the need for extensive patient followup, (2) the need for providers to learn more about community resources and how to use them, and (3) the well-being benefits providers receive from being able to offer non-pharmacological solutions (56). Such parallels suggest that arts/culture assets could be readily integrated with digital community-referral platforms to support health and healthcare. In addition, most platforms do not explicitly include arts/culture in their networks; as a result, piloting such inclusion would provide new, useful information regarding community health practices.
**8**.	Collect data from all participants, rather than strictly those being referred/prescribed.	Data collection for this study focused on CultureRx participants. However, the benefits or challenges of cultural participation are not limited to individuals receiving a healthcare-based referral. In the future, collecting data from *all* participants will generate more information regarding health outcomes–thus better informing providers' decisions to recommend an experience. In addition, asking everyone to provide feedback prevents singling out those who are present due to a referral, and may also bolster public awareness of the initiative. Of course, data collection should include a way to note how a given respondent heard about the program, so that data from referred participants can be extracted for analysis if necessary.
**9**.	Share data collection successes and tips.	Some CultureRx organizations learned successful processes for data collection, which improved participants' experience. Given that such learnings are likely transferable across organizations, they should be regularly shared. Similarly, when organizations encounter difficulties or unusual situations, they should be given opportunities to walk through their process with a program evaluator or, perhaps most helpfully, with similar organizations that may have helpful tips. As mentioned, organizations should also be urged and expected to use funds to enhance referral and data collection processes. Finally, as more arts-on-prescription programs are created and studied, evaluation methods and practices should be shared and analyzed to support improvement and synthesis.
**10**.	Address structural barriers to equity, access, and inclusion.	Initiatives such as CultureRx face equity challenges common to many other sectors and systems, including sustainability (short-term funding that expects long-term outcomes); culture (knowledgeability and representation regarding diverse communities served); and selection bias (organizations with the most access to competitive grants may not be the organizations most prioritized by communities). These concerns require intentional inquiry and action, as suggested by the following questions [quoted from Golden et al. (36)] • Is the organization or its programs in (or accessible to) marginalized communities? • What relationships do they have with such communities? • How is the voice of these communities informing the design and evaluation of the organization's programming? • How does the leadership's demographic make-up reflect that of the communities they are trying to reach? • How does the mission of participating programs and providers align with that of the overarching initiative?
**11**.	Implement frameworks for becoming antiracist and inclusive.	Several tools and frameworks exist for use by health service organizations to self-assess and prioritize areas for improvement on a continual basis [see (57–59)]. These standards and tools can also be used by arts/culture organizations to support them in increasing inclusivity of–and effectiveness for–diverse and marginalized communities.

## 7. Conclusion

The CultureRx initiative is the first program in the United States to offer arts and culture resources on prescription, and it comprises partnerships between 12 cultural organizations and 20 healthcare providers across the Commonwealth of Massachusetts. This evaluation examined the experiences of CultureRx participants, cultural organizations, and healthcare providers in order to identify barriers, successes, outcomes, and recommendations for short- and long-term development. Findings indicate that participants enjoyed the referred experiences, explicitly hoped to repeat them, and saw them as contributing to their wellbeing. Cultural organizations reported varied challenges as well as substantial learning. Providers saw the program as a new and critical addition to their toolkits; they also indicated that using CultureRx had a positive effect on their own wellbeing: a standout finding at a time when healthcare providers are experiencing unprecedented rates of burnout.

This pilot indicates that integrating arts/culture assets into health and social care approaches can enrich traditional models of community referral in the US to advance health and wellbeing. It offers a model not only for helping mitigate ill health through additional community assets, but also for actively cultivating positive wellbeing–which many providers, facilities, and referral networks are not yet set up to address. By including arts/culture resources when addressing social determinants of health, communities will be better positioned to equitably and holistically advance health.

## Data availability statement

The original contributions presented in the study are included in the article/supplementary material, further inquiries can be directed to the corresponding author.

## Ethics statement

The studies involving human participants were reviewed and approved by the Johns Hopkins Medicine IRB. Written informed consent from the participants' legal guardian/next of kin was not required to participate in this study in accordance with the national legislation and the institutional requirements.

## Author contributions

TG conceived of the study and this article, led the writing process, compiled sections, and drafted the discussion and recommendations. AML, NM, and MK contributed to initial codebooks and guides for analysis. TG, AML, AT, AM, TN, MM, NM, MK, and TB analyzed data and each wrote sections of the manuscript. AML, AT, AM, TN, MM, MK, and TG contributed to revisions. All authors have read and approved the final submission.

## References

[1] Magnan S. Social determinants of health 101 for health care: five plus five. NAM Perspectives. (2017) 7:10. 10.31478/201710c PMC8406598 34532697

[2] Whitman A De Lew N Chappel A Aysola V Zuckerman R Sommers BD. Addressing Social Determinants of Health: Examples of Successful Evidence-Based Strategies Current Federal Efforts. Washington, DC: Office of the Assistant Secretary for Planning Evaluation (2022). Available online at: https://aspe.hhs.gov/sites/default/files/documents/e2b650cd64cf84aae8ff0fae7474af82/SDOH-Evidence-Review.pdf (accessed August 25, 2022).

[3] Centers for Disease Control Prevention. Health in All Policies. AD for Policy and Strategy. CDC. (2016). Available online at: https://www.cdc.gov/policy/hiap/index.html (accessed August 1, 2022).

[4] Shattuck EC Perrotte JK Daniels CL Xu X Sunil TS. The contribution of sociocultural factors in shaping self-reported sickness behavior. Front Behav Neurosci. (2020) 14:4. 10.3389/fnbeh.2020.00004 32038193 PMC6992553

[5] Fancourt D Steptoe A. The art of life and death: 14 year follow-up analyses of associations between arts engagement and mortality in the English longitudinal study of ageing. The BMJ. (2019) 18:367. 10.1136/bmj.l6377 31852659 PMC7190042

[6] Mak HW Coulter R Fancourt D. Associations between neighborhood deprivation and engagement in arts, culture and heritage: evidence from two nationally-representative samples. BMC Public Health. (2021) 21:1–10. 10.1186/s12889-021-11740-6 34530782 PMC8444412

[7] White MP Elliott LR Grellier J Economou T Bell S Bratman GN. Associations between green/blue spaces and mental health across 18 countries. Sci Rep. (2021) 11:1–12. 10.1038/s41598-021-87675-0 33903601 PMC8076244

[8] Ducharme J Wolfson E. How Your Zip Code Could Affect Your Lifespan. Time. (2019).

[9] Schwarz D. Data Provides a Deeper Understanding of Life Expectancy Gaps - RWJF. New Jersey, NJ: Robert Wood Johnson Foundation. (2018).

[10] Hall RL Jacobson PD. Examining whether the health-in-all-policies approach promotes health equity. Health Aff. (2018) 37:364–70. 10.1377/hlthaff.2017.1292 29505382

[11] Rudolph L Caplan J Ben-Moshe K Dillon L A. Guide for State and Local Governments. Washington, DC: American Public Health Association and Public Health Institute (2013).

[12] Hand J Sherman D Bullock M. The Role of Arts Culture in Equitable Community Development: A Visual Analysis. ArtPlace America (2020). Available online at: https://creativeplacemakingresearch.org/

[13] Pastor M Terriquez V Lin M. How community organizing promotes health equity, and how health equity affects organizing. Health Affairs. (2018) 37:358–63. 10.1377/hlthaff.2017.1285 29505367

[14] Sonke J Golden T Francois S Chandra A Clemmons L Fakunle D. Creating Healthy Communities: Art Public Health in America. Gainesville, FL: University of Florida Center for Arts in Medicine and ArtPlace America (2019).

[15] Corburn J. Street science: Local knowledge and environmental justice. In: Tackling Health Inequities Through Public Health Practice: Theory To Action. (2010). p. 417.

[16] Jackson MR. Addressing inequity through public health, community development, arts, and culture: confluence of fields and the opportunity to reframe, retool, and repair. Health Prom Prac. (2021) 22:141S−6S. 10.1177/1524839921996369 33942651

[17] University of Florida Center for Arts in Medicine. Advisory Briefs, Arts in Public Health Resources, Creating Healthy Communities: Arts + Public Health in America. (2019). Available online at: https://arts.ufl.edu/sites/creating-healthy-communities/resources/advisory-briefs/ (accessed August 1, 2022).

[18] Husk K Elston J Gradinger F Callaghan L Asthana S. Social prescribing: where is the evidence? Br J Gen Prac. (2019) 69:6–7. 10.3399/bjgp19X700325 30591594 PMC6301369

[19] Buck D Ewbank L. What is Social Prescribing? The King's Fund. (2020).

[20] Chatterjee HJ Camic PM Lockyer B Thomson LJM. Non-clinical community interventions: a systematised review of social prescribing schemes. Arts Health. (2018) 10:97–123. 10.1080/17533015.2017.1334002

[21] Mckenzie K Karen R Murray K. Which elements of socially prescribed activities most improve wellbeing? Nurs Times. (2021) 117:39–41.

[22] Thomson LJ Camic PM Chatterjee HJ. Social Prescribing: A Review of Community Referral Schemes. London: University College London (2015).

[23] Morse DF Sandhu S Mulligan K Tierney S Polley M Chiva Giurca B. Global developments in social prescribing. BMJ Glob Health. (2022) 7:e008524. 10.1136/bmjgh-2022-008524 35577392 PMC9115027

[24] Jensen A Stickley T Torrissen W Stigmar K. Arts on prescription in Scandinavia: a review of current practice and future possibilities. Pers Pub Health. (2016) 137:268–74. 10.1177/1757913916676853 27852837

[25] Visanich V Attard T. Towards the social prescription of the arts: The arts in health and social care in Malta. J Appl Arts Health. (2021) 12:163–76. 10.1386/jaah_00065_1

[26] Zurynski AY Vedovi A Smith KL. Social Prescribing : A Rapid Literature Review to Inform Primary Care Policy in Australia. NHMRC Partnership Centre for Health System Sustainability Australian Institute of Health Innovation. Macquarie: Macquarie University (2020), 1–24.

[27] Lindau ST Makelarski JA Abramsohn EM Beiser DG Boyd K Chou C. Community Rx: A Real-World Controlled Clinical Trial of a Scalable, Low-Intensity Community Resource Referral Intervention. Washington, DC: American Public Health Association Inc. (2019). p. 600–6. 10.2105/AJPH.2018.304905 PMC6417580 30789775

[28] Iott BE Eddy C Casanova C Veinot TC. More than a database: understanding community resource referrals within a socio-technical systems framework. In: AMIA Annual Symposium Proceedings 2020, Vol. 2020. American Medical Informatics Association (2020). p. 583. 33936432 PMC8075446

[29] Norman GJ Wade AJ Morris AM Slaboda JC. Home and community-based services coordination for homebound older adults in home-based primary care. BMC Geriatr. (2018) 18:1–9. 10.1186/s12877-018-0931-z 30305053 PMC6180527

[30] Thomas-Henkel C Schulman M. Screening for Social Determinants of Health in Populations with Complex Needs: Implementation Considerations. Trenton, NJ: Center for Health Care Strategies, Inc. (2017).

[31] Academy of Family Physicians A. *Social Determinants of Health Guide to Social Needs Screening Tool and Resources*. The EveryONE Project TM (2018). Available online at: https://www.aafp.org/family-physician/patient-care/the-everyone-project/toolkit.html

[32] Schroeder S. Utilization of community resources. South Dakota Med. (2017) 2:59–61. 28817865

[33] Christensen K Soucie J Scholle SH. Implementing a Community Referral Platform: Recommendations From a Real-World Implementation Experience Qualitative Findings December 2020 National Committee for Quality Assurance (NCQA). (2020). Available online at: www.ncqa.org (accessed August 1, 2022).

[34] Sherry M Blumgart M Rosen M. Building Healthier Communities: A Community Action Framework. Unite Us (2019). Available online at: https://uniteus.com/report/building-healthier-communities-a-community-action-framework/

[35] Geisler C Swarts J. Coding Streams of Language. The WAC Clearinghouse. Boulder, CO: University Press of Colorado. (2019).

[36] Golden TL Springs S Kimmel HJ Gupta S Tiedemann A Sandu CC. The use of music in the treatment and management of serious mental illness: a global scoping review of the literature. Front Psychol. (2021) 12:880. 10.3389/fpsyg.2021.649840 33868127 PMC8044514

[37] Fancourt D Finn S. What is the Evidence on the Role of the Arts in Improving Health and Well-Being? A Scoping Review. Geneva: World Health Organization (2019). 32091683

[38] Pesata V Colverson A Sonke J Morgan-Daniel J Schaefer N Sams K. Engaging the arts for wellbeing in the united states of America: a scoping review. Front Psychol. (2022) 22:6524. 10.3389/fpsyg.2021.791773 35222154 PMC8863598

[39] Smith KN Cullinan D Douglas P Erickson D Imah S Jackson A. Arts & Public Health: Core Outcomes Set Briefing Paper. Gainesville, FL: University of Florida Center for Arts in Medicine (2021).

[40] Brandling J House W. Investigation Into the Feasibility of a Social Prescribing Service in Primary Care: A Pilot Project. Repository for Arts Health Resources. (2007). Available online: https://www.artshealthresources.org.uk/docs/investigation-into-the-feasibility-of-a-social-prescribing-service-in-primary-care-a-pilot-project/ (accessed November 2, 2022).

[41] Husk K Blockley K Lovell R Bethel A Bloomfield D Warber S. What approaches to social prescribing work, for whom, and in what circumstances? A protocol for a realist review. Syst Rev. (2016) 5:1. 10.1186/s13643-016-0269-6 27255362 PMC4891904

[42] American Hospital Association. Data Brief: Health Care Workforce Challenges Threaten Hospitals' Ability to Care for Patients. (2021). Available online at: www.aha.org (accessed August 1, 2022).

[43] Frogner BK Dill JS. Tracking turnover among health care workers during the COVID-19 pandemic. JAMA Health Forum. (2022) 3:e220371. 10.1001/jamahealthforum.2022.0371 35977315 PMC8994131

[44] Nishimura Y Miyoshi T Hagiya H Kosaki Y Otsuka F. Burnout of healthcare workers amid the COVID-19 pandemic: a Japanese cross-sectional survey. Int J Environ Res Public Health. (2021) 18:1–8. 10.3390/ijerph18052434 33801349 PMC7967555

[45] Willard-Grace R Knox M Huang B Hammer H Kivlahan C Grumbach K. Burnout and health care workforce turnover. Ann Fam Med. (2019) 17:36–41. 10.1370/afm.2338 30670393 PMC6342603

[46] Martin L Oepen R Bauer K Nottensteiner A Mergheim K Gruber H. Creative arts interventions for stress management and prevention-a systematic review. Behav Sci. (2018) 8:2. 10.3390/bs8020028 29470435 PMC5836011

[47] Koch SC Riege RFF Tisborn K Biondo J Martin L Beelmann A. Effects of dance movement therapy and dance on health-related psychological outcomes. A meta-analysis update. Front Psychol. (2019) 10:1806. 10.3389/fpsyg.2019.01806 31481910 PMC6710484

[48] Lee KC Chao YH Yiin JJ Hsieh HY Dai WJ Chao YF. Evidence that music listening reduces preoperative patients' anxiety. Biol Res Nurs. (2012) 14:78–84. 10.1177/1099800410396704 21278165

[49] Nilsson U. The anxiety- and pain-reducing effects of music interventions: a systematic review. AORN J. (2008) 87:780–807. 10.1016/j.aorn.2007.09.013 18395022

[50] Uttley L Scope A Stevenson M Rawdin A Taylor Buck E Sutton A. Systematic review and economic modelling of the clinical effectiveness and cost-effectiveness of art therapy among people with non-psychotic mental health disorders. Health Technol Assess. (2015) 19:1–120. 10.3310/hta19180 25739466 PMC4781197

[51] Xurui T Yaxu Y Qiangqiang L Yu M Bin Z Xueming B. Mechanisms of creativity differences between art and non-art majors: a voxel-based morphometry study. Front Psychol. (2018) 9:2319. 10.3389/fpsyg.2018.02319 30618898 PMC6301215

[52] Wheatley D Bickerton C. Measuring changes in subjective well-being from engagement in the arts, culture and sport. J Cult Econ. (2019) 43:421–42. 10.1007/s10824-019-09342-7

[53] Association for Behavioral Health. Outpatient Mental Health Access and Workforce Crisis Issue Brief. Washington, DC: Association for Behavioral Health (2022).

[54] Coombs NC Meriwether WE Caringi J Newcomer SR. Barriers to healthcare access among U. S adults with mental health challenges: a population-based study SSM. Popul Health. (2021) 15:100847. 10.1016/j.ssmph.2021.100847 34179332 PMC8214217

[55] Price V. Mass Kids in Psychiatric Crises Forced to Spend Days, Weeks in ERs Waiting for help – Boston News, Weather, Sports .WHDH 7News. 7 News Boston. (2022). Available online at: https://whdh.com/news/mass-kids-in-psychiatric-crises-forced-to-spend-days-weeks-in-ers-waiting-for-help/ (accessed May 17, 2022).

[56] Kung A Cheung T Knox M Willard-Grace R Halpern J Olayiwola JN. Capacity to address social needs affects primary care clinician burnout. Ann Fam Med. (2019) 17:487–94. 10.1370/afm.2470 31712286 PMC6846269

[57] Association of American Medical Colleges. Anti-Racism Resources. Association of American Medical Colleges. (2022). Available from: https://www.aamc.org/about-us/equity-diversity-inclusion/anti-racism-resources (accessed August 1, 2022).

[58] California Improvement Network CHF. A Toolkit to Advance Racial Health Equity in Primary Care Improvement CIN. Oakland, CA: California Improvement Network CHF. (2022).

[59] Society for Adolescent Health Medicine. Anti-Racism toolkit. Society for Adolescent Health and Medicine. (2022). Available online at: https://www.adolescenthealth.org/Resources/Anti-Racism-Toolkit.aspx (accessed August 1, 2022).

